# Determination of the Experiences of Patients Transferred from the Intensive Care Unit to the Ward

**DOI:** 10.3390/healthcare13080945

**Published:** 2025-04-20

**Authors:** Pinar Tekinsoy Kartın, Dilek Bozot Kayasan, Ülkü Özdemir

**Affiliations:** 1 Faculty of Health Sciences, Department of Internal Medicine Nursing, Erciyes University, 38260 Kayseri, Turkey; ozdemir.ulku@erciyes.edu.tr; 2 Vocational School of Health Services, Anesthesiology Department, Aksaray University, 68100 Aksaray, Turkey; dilekbozotkayasan@aksaray.edu.tr

**Keywords:** intensive care, patient experience, anxiety, spiritual care

## Abstract

Introduction: Patients in intensive care units (ICUs) face factors that cause anxiety, fear, pain, depression, and adverse health behaviors. This qualitative study aims to determine patients’ experiences when transferred from the ICU to the ward. Methods: Thirteen individuals who were transferred from the ICU to the ward were included in this study. Interviews were conducted using a face-to-face method in the patient’s room. The interviews were recorded with a voice recorder with the consent of the patients. Codes, categories, and themes were created, and content analysis and descriptive analysis were carried out after the audio recordings were converted into text. Results: Patients reported receiving adequate physical and personal care in the ICU and were satisfied with its continuity. They felt safe due to the close attention of healthcare professionals and continuous treatment. Although they received psychological and social support from nurses, they were negatively affected by constant lights, patient noises, and nursing conversations. Patients experienced anxiety about not knowing the health status and time of day, about their relatives, their homes, and other critically ill patients in intensive care. Some patients reported fear of not being able to leave the intensive care unit, relapse, disability, or death. Patients reported pain due to the cold environment, lighting, probes, drains, and positioning. Patients suggested that healthcare personnel communicate better with them, have a clock they can see, reduce noise, and have caregivers of the same gender. They emphasized the need for moral support. Conclusions: Constant light in the intensive care unit, sounds from other patients, nurses talking among themselves, not being able to see their relatives, not knowing what time of day it is, and wondering caused anxiety in the patients. It was determined that patients experienced pain due to catheter, drain, aspiration procedures, cold environment, and position in bed. Notably, patients reported that they needed moral support and wanted to receive care from caregivers of the same gender.

## 1. Introduction

Intensive care units are special treatment units that provide treatment and care with technological and biomedical devices developed for the follow-up, treatment, and care of life-threatening organ failures due to acute and chronic diseases [1].

Patients encounter many stressors, both physical and psychosocially, in ICUs [1]. In the intensive care setting, patients suffer from many psychosocial problems because of being monitored and connected to ventilator support, being bedridden due to urinary catheters and infusion sets, exposure to the sound of biomedical devices, constant exposure to light, unpleasant images, odors and painful interventions, and communication difficulties. Patients also face problems such as conditions requiring treatment in the ICU, isolation, disruption of the day–night concept and sleep patterns, unknown environment, not being able to see family members, and not being able to receive sufficient information from healthcare personnel about treatment, disease process, and interventions [2,3,4]. In addition, it is reported that patients experience moderate to severe pain resulting from invasive procedures, trauma, long-term inactivity, routine nursing care, and existing illness, which causes negative mental and physical effects, unmet patient needs, and isolation of the patient [5].

The physical environment of intensive care units (ICUs) significantly impacts patients’ sleep patterns, stress levels, and overall physical comfort. Factors such as constant lighting, noise of medical equipment and conversations, and the presence of various medical devices contribute to sleep disturbances and increased stress among ICU patients [6]. Maintaining an appropriate temperature in ICUs is crucial for patient comfort and recovery. It has been reported that significant stressors affecting patients’ comfort according to patients were environmental factors such as room temperature and bed positioning [7]. Additionally, research has also indicated that improper temperature regulation and patient positioning may lead to discomfort and sleep disturbances in ICU settings [8]. To address these issues, it is recommended to maintain ICU temperatures between 20 and 24 °C (68–75 °F) with relative humidity levels of 30–60%, as these conditions have been associated with improved patient comfort and outcomes [9]. Addressing these environmental factors is crucial for improving patient outcomes and promoting recovery in ICU settings [4,10]. Admission to ICUs is associated with significant psychological distress, including symptoms of anxiety and depression. Studies have documented that ICU patients often experience post-traumatic stress disorder (PTSD), anxiety, and depression during their recovery period [11]. Çam and Şahin have also determined moderate anxiety and depression in ICU patients [12]. Implementing systematic screening for these conditions and providing appropriate psychological support are essential to comprehensive ICU care [13]. In one study, 72% of patients were found to be at risk of depression and 42% were at risk of anxiety. This has a negative impact on the general well-being of patients [14]. Intensive care unit patients often face significant challenges in communication due to mechanical ventilation, leading to psychological issues such as anxiety, fear, and depression. The inability to speak and express needs may cause moderate to extreme psycho-emotional distress [15]. It has been reported in another study that critically ill patients were often disturbed by the physical environment of the ICU, such as being bedridden, lack of a television and radio, limited visiting hours, and seeing patients whose condition is serious or deteriorating [4]. It is stated that patients experience basic psychosocial problems such as anxiety, depression, and post-traumatic stress disorder even after they are discharged from the ICU [16].

The aim of care in ICUs is to physically and psychosocially support the patient and his/her family and to allow patients to be discharged with positive experiences in ICUs in addition to saving lives [17]. Qualified nurses ensure that the physical, psychological, and social care needs of patients receiving treatment in the ICU are met and that they have a positive intensive care experience [18].

It is thought that an in-depth questioning of the factors and experiences that cause anxiety, fear, pain, depression, and negative health behaviors during the critical periods of patients in the ICU will guide healthcare professionals in determining care needs and in planning appropriate interventions. Although there are many quantitative studies examining the experiences of intensive care patients, there are few qualitative studies in the literature [1,3,5,12,14,19,20].

The aim of this study is to determine the intensive care experiences of patients transferred from the intensive care unit to the ward.

## 2. Methods

### 2.1. Study Design

This study was carried out in a phenomenology design among qualitative research methods to determine the experiences of patients who were transferred from the ICU to the ward. Phenomenology is a qualitative research design that investigates a person’s lived experiences of a phenomenon. The aim of this design is to inform the participants about the phenomenon being researched [21].

### 2.2. Study Group

Criterion sampling, which is a type of purposive sampling, was used in this study. Accordingly, patients, who were transferred from the ICU to the ward in a research hospital in the Central Anatolia Region, were selected among individuals who were suitable given the purpose of this study by the researchers. This study included patients who received care and treatment in intensive care units for at least 24 h, were 18 years of age or older, had no hearing or visual impairment, did not have neurological or psychiatric diseases, could speak Turkish, and agreed to participate in this study. Patients who refused to be interviewed or audio-recorded or who wished to leave the study during the interview were not included in this study.

The 13 patients interviewed were coded as P1, P2, P3, etc. The characteristics of the participants were determined in the presence of demographic questions asked during the interview. Information about the participants is shown in Table 1.

### 2.3. Data Collection Tools

Data were collected by the researchers with the “Patient Information Form” [3] and with the “Semi-Structured Interview Form” [3,4,5], which were created in line with the literature and included 7 questions about socio-demographic characteristics and another 7 questions about characteristics of the disease. Two experts’ opinions were taken before applying the interview form. Necessary corrections were made to the questions in line with the experts’ feedback, and 13 research questions were formed which questioned adequate physical care, psychosocial support, environmental factors, feeling safe, factors increasing anxiety and pain, communication, stress, and spiritual care.

### 2.4. Data Collection Process

The data were collected by a researcher between the dates of 1 April 2022 and 30 July 2022 with the patients transferred from the ICU to the internal and surgical adult inpatient clinics, at 08.00 a.m.–16.00 p.m., and outside the treatment time, within the period determined with the patient. The data were collected by the face-to-face in-depth interview method. Face-to-face interviews were recorded in a quiet environment in the patient’s room, both by writing and with a voice recorder, after voluntary consent was obtained from the patient. The interview with each patient lasted an average of 25–30 min. The recorded interviews were then listened to, and the answers were transcribed and analyzed. Fifteen patients were interviewed to collect the data. The data of two patients with a diagnosis of COPD who spoke intermittently due to shortness of breath during the interview were not included in this study. This study was completed with a total of 13 patients who provided sufficient data saturation.

### 2.5. Ethical Dimension of the Research

Ethics committee approval (ethics committee number: 2022/219) from the non-pharmaceutical clinical research ethics committee of the university and institutional permission from the institution where this study was conducted were obtained. Written and verbal consent were obtained from the patients by signing an informed consent form. In this study, patient names were specified in code as P1, P2, P3, etc., in line with the ethical rules.

### 2.6. Validity and Reliability

The interview form applied by the researcher was presented to two different experts to obtain their opinions in order to increase the internal validity of this study. In line with the opinions of the experts, the form was rearranged in terms of the intelligibility and adequacy of the questions.

The research model, study group, data collection tools, data collection, data analysis, and organization of the findings were described in detail to increase transferability. The purposive sampling method was used to select participants that fit the purpose of this study. In addition, another factor that increases external validity is that the number of participants is 13.

The subject and themes were presented to the experts for their opinions, and consistency was ensured. The findings obtained from the interview questions were also presented to the reader without comment. The interviews were recorded on a voice recorder and were played to the participants after the recording had finished. We attempted to prevent data loss, and the internal reliability of the research was increased. The findings were presented to the experts for their opinions by the researcher, detailed in Section 4, Section 5 and Section 6, to increase the external reliability, ensuring the confirmability of the research.

### 2.7. Data Analysis

In this study, both content analysis and descriptive analysis were used to transcribe and analyze the records obtained from the semi-structured interviews to reveal previously unclear views and scopes. Codes were made by the researcher in line with the data obtained from the interviews. Categories and a single theme were created from the codes. These were presented to the expert, and the data were classified under themes and categories and made to be meaningful for the reader as a result of the expert opinion. The question titles in the Findings Section constitute the theme of “intensive care experiences of the patients”.

## 3. Findings

The intensive care experiences of the patients were given under a single theme. Sixteen categories were discussed under this theme. The answers of the patients regarding their experiences during the treatment process in the ICU were presented by creating categories.

Categories included physical care, receiving psychological and social support, environmental factors, feeling safe, factors that increase anxiety, topics of interest, the thought of getting out of the ICU or not, factors that increase pain, communication with a healthcare professional, suggestions about communication, suggestions about care, effective and ineffective methods to cope with stress, and thoughts about spiritual care (Table 2).

**Patients reported that their physical and personal care (such as personal hygiene, sheet changes, and shaving) were adequately provided and satisfied.**

“They provided excellent care, wiped me with cologne, and washed me with alcohol… I didn’t need a shave, but if I did, they would have shaved me… The sheets were spotless. If there was even a tiny drop of blood, they changed them immediately”.

(P5)

“I vomited, and that gray-haired nurse cleaned my face, changed my clothes and sheets, and wiped my chest. They are perfect. A patient lay down, and their armpits were very dirty; they cleaned them. The nurses are excellent”.

(P6)

**Patients reported receiving psychological and social support from doctors and nurses. However, one patient mentioned hearing nonexistent sounds and expressed a desire for psychological support.**

“The doctors and nurses reassured me that I would be fine. They said I would feel better once I was transferred to the ward. Every morning, they greeted me with ’Good morning’ and asked how I was doing”.

(P3)

“My relatives came from Istanbul, and they arranged for me to see them. That was psychological support; it boosted my morale”.

(P4)

“The nurses played music for me, and it helped. The music stabilized my blood pressure—it lifted my spirit and brightened my face”.

(P8)

“The doctors and nurses brought me a TV and set it up. I was feeling bored, and the TV provided great support”.

(P12)

“I would have liked to receive psychological support from a specialist because I started hearing strange sounds there. I was surprised to hear the distant background music as if coming from nowhere”.

(P10)

**Patients reported being negatively affected by the lights, the noises from other patients, and the conversations among nurses throughout the night.**

“Patients scream, and you wake up immediately—even though you’re already having trouble sleeping… You can’t adjust the lights yourself. Nurses perform care at 2 AM and must turn the lights on. The nurses talk, someone comes to put in an IV, and you wake up. You wake up to everything. And I had a machine next to me—if I lifted my arm, the IV tube would bend, and the alarm would keep beeping. The noise was nerve-wracking”.

(P3)

“I don’t like noise, so I was disturbed by the nurses talking among themselves”.

(P8)

“My eyes became blurry; I couldn’t see because of the lights”.

(P1)

“The light affected me a lot. I don’t like light; it made me sleepless”.

(P13)

**Patients reported feeling safe due to the high level of attention from healthcare professionals and the constant medical care.**

“Nurses and doctors were coming and going all the time. Even when nurses were sitting at their desks, they kept an eye on me to see if I needed anything. May God bless them”.

(P12)

“You feel safer than at home because you are constantly being treated, and everyone wants you to get better. Plus, different doctors kept checking on me, so of course, I felt safe”.

(P3)

“Once a patient lies down, they immediately take care of them. Sometimes 10 people rush in at once to save you from death. That gave me confidence”.

(P5)

However, one patient felt unsafe due to the noise.

“I never felt safe. I was disgusted by the noise. People who had undergone angioplasty were brought there, screaming, yelling, and crying”.

(P4)

**The factors that increased patients’ anxiety the most were not being able to see their relatives, being unclothed, fear of disease progression, thoughts of not recovering, fear of relapse, disability, and death.**

“My family left me, and I wondered why they put me in intensive care. I got stressed. They told me that nobody is allowed in the ICU. Being naked was embarrassing; I felt ashamed in front of the men. When I was first admitted, I thought I was going to die. People say, ‘Whoever enters the ICU dies’, I kept thinking about that”.

(P1)

“Will my illness come back? Will it get worse? Will I have an embolism again? Will I have breathing problems and be put on a machine? Will I be able to leave here alive? I also know that intubation can cause memory loss and paralysis. I still worry about whether I’ll have any lasting effects”.

(P10)

“I recited the Shahada (Islamic declaration of faith), repented for my sins, and told my mother to forgive me, saying, ‘I’m not well’”.

(P8)

“When my relative visited, food arrived. I asked if they could feed me, but the nurse said, ‘I have to do it’. It would have been a morale boost if my relative had fed me for 10 min. Also, I didn’t want a male nurse to insert a catheter for me”.

(P10)

**Patients were most curious about their loved ones, their homes, the health conditions of other ICU patients, the time of day, and the unknown aspects of their surroundings.**

“I thought about and worried about my family, my land, my garden, and my children”.

(P11)

“A clock would have been useful. I kept asking the nurses for the time. I didn’t know what time it was at night. Maybe there was a clock on the monitor, but I wasn’t aware of it”.

(P8)

“I kept asking what happened to the other patients next to me and what was wrong with them”.

(P3)

“The machines there were impressive. When the nurse was suctioning a patient’s throat with a tube, I was curious and asked what they were doing”.

(P7)

**Patients reported experiencing pain due to the cold environment, the lights, catheters, drainage tubes, aspiration procedures, and the position they were lying in.**

“The tube in my throat caused a little pain and burning, and it also affected my voice. Lying in the same position made my back ache. The catheter hurt, but I felt relieved once it was removed. The lights gave me a headache”.

(P13)

“The cold environment made my stomach ache. Because I was cold, I felt as if my heart was beating inside my lungs, causing deep pain”.

(P8)

“They put a tube in my throat—it hurt… I coughed up blood”.

(P4)

“They put a tube in my mouth and taped it. Then I couldn’t speak. My throat still hurts; look, my voice is gone”.

(P10)

**Patients stated they wanted healthcare professionals to provide information about medical procedures and devices.**

“One doctor never communicated with me, didn’t explain anything. I would have liked an explanation”.

(P2)

“I had an endoscopy in the morning. Then they took me to the ward. I wasn’t given any information for four hours, and during that time, I was anxious and stressed”.

(P9)

**Patients expressed a desire for spiritual support during their ICU stay.**

“I would have liked spiritual care in the ICU, for prayers to be recited. That would have comforted me… I felt guilty for not being able to pray”.

(P4)

“If a religious leader came and prayed, I would have listened. It would have calmed me”.

(P7)

“I would have liked to receive spiritual care in the ICU. Talking about religion brings peace”.

(P10)

## 4. Discussion

In our study, most of the patients reported that they received adequate physical and personal care, such as personal cleaning, bed changing, and shaving carried out continuously, and they were satisfied with this situation. It has been reported in the literature [1,16,22,23] that patients treated in the ICU received adequate physical care and had positive experiences and high satisfaction levels, which support our study findings. Negative intensive care experience and low levels of satisfaction have also been reported by patients treated in the ICU in several studies [12,17,22,24]. The personal care of patients in the intensive care unit (ICU) constitutes a fundamental component of basic nursing care, routinely provided by nurses. Additionally, nurses, in collaboration with auxiliary health personnel, regularly perform bed changes to ensure patient comfort and hygiene. Consequently, the findings of this study suggest that patients reported a high level of satisfaction with the care received.

The patients in our study mentioned that they received sufficient psychological and social support from the physicians and nurses while meeting with their families and that they were provided additional psychological support. Although previous studies have shown that patients face communication difficulties in the ICU, the findings show that they often experience identity loss and have difficulty communicating due to uncertainty about when they will regain their ability to speak. Furthermore, communication in the ICU is often poor and limited to basic issues [22,25]. The difference in our study findings may result from the fact that healthcare staff were able to communicate with patients since non-intubated patients were more involved in this study.

A lack of communication with patients in the intensive care environment causes both anxiety and prolongs the recovery period when patients are transferred from the ICU to the ward. So, the cost of care increases while the quality of care decreases. Whether patients are conscious or not, nurses should provide verbal and nonverbal communication and include patients in their care when necessary [26].

Noise, alarms, staff sounds, medical machines, light, exposure to excessive stimuli, lack of analgesia, monitoring vital signs, changing the position of patients, sedentariness, room temperature, and disruption of the sleep cycle in ICUs are listed as the causes of anxiety and agitation in patients [27].

In our study, the patients were highly affected by the lights being constantly on at night and disturbed by the voices of other patients and the conversations between nurses, which then increased their anxiety levels.

Studies similar to our study reported that alarm sounds, speech sounds, telephone ringing sounds, oxygen treatment sounds, radio–television sounds, other patients’ voices, and repair sounds caused noise and disturbed patients, meaning that they had trouble sleeping due to these sounds [19,28], and they were often afraid of the noises [22]. Noise in the intensive care unit is an important factor causing sleep deprivation.

Reducing noise and light during sleep periods in the ICU is an essential intervention for the comfort of the patients.

Patients reported feeling safe because the healthcare staff were involved, and they were under constant surveillance. Like our study results, most of the patients felt safe due to seeing their family and relatives and the sincere behaviors of the nurses in the relevant studies [29]. If patients in intensive care are away from their families and feel lonely, the care and assistance of medical personnel may have contributed to their psychological well-being.

The factors that most increased the anxiety and aroused the curiosity of the patients in this study were not seeing their relatives and worrying about the health status of other patients.

Other studies conducted report that patients experience not being able to see their families and spouses in the hospital [14,30,31] and a feeling of abandonment by their family/caregiver [30,31].

The results of this study suggest that due to the restriction of visits in intensive care units, patients are separated from their families and potentially experience feelings of isolation and distress, which increases their anxiety.

Studies have reported that patients in intensive care units have difficulty distinguishing between day and night and therefore experience nightmares and hallucinations, and this causes delirium [16,22]. In this study, the patients reported that they could not distinguish between day and night.

The incidence of delirium is approximately 14% to 24% in the general medical unit and 70% to 87% in the intensive care unit. Delirium has serious consequences for patients during their hospital stay (increased mortality and prolonged hospital stay) and after discharge (long-term cognitive impairment). Communicating with patients, enabling patients to talk to their families, and reminding patients about the concepts of time and place are essential nursing interventions in preventing delirium and hallucinations in the intensive care setting [32].

In this study, it was observed that some patients experienced fear of not being able to recover and not being able to leave the intensive care unit, fear of recurrence of their disease, disability, and death. In the study of Chahraoui et al. (2015), it was found that patients experienced feelings of sadness, fear, and approaching death related to staying in the ICU [31]. In another study, it was determined that patients did not know how long they would stay in intensive care and experienced fear of death [33]. Although the patients who participated in this study reported that they received support from doctors and nurses and were satisfied with the care shown to them, this result may be due to reasons such as not receiving sufficient information from healthcare personnel about the treatment process and their illness, uncertainty about the prognosis of the disease, and fear of death due to the intensive care environment.

The patients in this study reported that they experienced pain due to catheters, drains, aspiration procedures, and the position they were in.

It is reported in the literature that patients in intensive care experience alarming pain [22,26,30,31].

It is an expected problem that patients experience pain due to the position of their long-term hospitalization and the interventional procedures performed. Therefore, it is important to assess the level of pain that may develop in patients and to plan appropriate nursing interventions.

In this study, it was determined that pain, inability to sleep, being away from family, talking loudly, and not being able to distinguish day and night, as well as patients being naked and being cared for by healthcare personnel of different genders, cause stress. Studies have reported that medical interventions and care practices performed in the intensive care environment, not being able to distinguish between day and night, not having family members around, healthcare personnel talking loudly, men and women staying in the same room, a lack of privacy, and the environmental conditions of the intensive care unit cause stress for patients [20,30,33,34].

The fact that patients in intensive care have a serious illness, have limited contact with family, have unavoidable environmental risks such as noise, light, and sound, and are unable to distinguish between day and night may have caused stress due to reasons such as the patients not being accustomed to it, and also the fact that patients are naked in the intensive care unit and are cared for by healthcare personnel of different genders due to the religious beliefs of individuals in the community where this study was conducted.

Nurses working in intensive care units should be aware of the factors that cause stress in patients, be able to assess stress, and plan care interventions. They should also know pharmacological and non-pharmacological methods of stress management. Nurses can include non-pharmacological approaches such as music, aromatherapy, massage, and spiritual care support [35]. Nurses should be careful to provide care that is compatible with patients’ religious beliefs while providing care. It may be recommended that devices such as curtains and screens be used to protect patients’ privacy.

This study determined that patients wanted spiritual support during the intensive care process, such as reading prayers and conversations with religious figures. In Türkiye, spiritual support services are provided in hospitals based on the patient’s preferences. However, this study shows that patients’ needs for spiritual support mean that not enough support is provided.

This study found that patients wanted spiritual support during intensive care, such as prayer and clergy conversations. Spiritual support services are provided in hospitals in Türkiye according to the patient’s preference. However, this study shows that patients need spiritual support because spiritual support is not provided sufficiently.

Therefore, nurses in intensive care units (ICUs) should systematically assess and address the spiritual care needs of patients, as spirituality plays a crucial role in coping with illness and distress. Patients in critical conditions may experience heightened emotional vulnerability and seek moral and emotional support from religious officials to find comfort and meaning in their experiences. Integrating spiritual care into patient-centered nursing practices can contribute to overall well-being, reduce anxiety, and enhance the quality of care provided in the ICU setting.

## 5. Limitations

Since this research is a qualitative study, the findings may not be generalizable to all ICU patients and other healthcare settings due to the limited number of patients due to the nature of this study and the fact that the research was conducted in a single center. Patients’ experiences and perceptions were based on self-reports, which may be influenced by memory bias or emotional state. Some ICU patients may have cognitive issues due to sedation, medications, or illness, affecting their ability to recall and report their experiences accurately. Differences in patients’ health conditions, severity of illness, and treatments may also impact their experience, making it difficult to generalize the results.

## 6. Conclusions

According to the study results, it was determined that patients in intensive care units are generally satisfied with the physical and personal care and support received from healthcare professionals. However, they experience stress due to environmental factors such as light, noise, sound, recurrence of the disease, fear of death, and separation from family members and psychological factors such as lack of privacy. It was concluded that patients need spiritual and emotional support. In order to improve the ICU experience, it is important to minimize environmental stress factors to increase patient comfort, improve communication between healthcare personnel and patients, and provide psychological and spiritual support. Being aware of these factors and implementing appropriate interventions can reduce anxiety and stress, improve coping mechanisms, and increase the general well-being of ICU patients. Providing nursing care with an individualized, holistic approach; minimizing noise and excessive lighting to increase patient comfort; structuring the treatment plan in a way that does not disrupt patients’ sleep patterns; respecting personal preferences while providing care to patients; determining patients’ religious and spiritual care needs and bringing them together with experts and clergy in this field; taking care to bring patients together with their families during visiting hours; reminding patients of the time and having a clock in a visible place so that they can distinguish day from night; and providing patients with the opportunity to listen to music and watch movies using devices such as a television, tablet, and music system are all recommended.

## 7. Implications for Clinical Practice

- This study captures firsthand experiences of ICU patients, providing valuable insights into their physical, psychological, and emotional well-being.
- It examines multiple aspects of ICU care, including physical comfort, emotional support, communication, and environmental factors.
- This study highlights common challenges patients face, such as noise, lighting, and psychological stress; improving these challenges could help improve ICU care.
- Patients’ suggestions for improving ICU conditions (e.g., better communication, reduced noise, time awareness) can guide healthcare professionals in enhancing patient care.
- By addressing emotional and psychological concerns, this study emphasizes the importance of holistic care in ICU settings.
- The findings on stress management strategies provide valuable information on how patients cope with ICU-related stress and what support they need.
- This study highlights the need for individualized care and better patient–healthcare provider communication to improve the ICU experience.
- A multi-center approach review, where the experiences of intensive care patients are learned, is warranted in future research.
- The results of this study will likely serve as a resource for meta-synthesis studies.

## Figures and Tables

**Table 1:** Introductory characteristics of the participants.

Participant	Gender	Age (Years)	Occupation	Marital Status	Educational Status	Diagnosed with	ICU Length of Stay (Days)
P1	Female	82	Housewife	Single	Illiterate	COPD	3
P2	Male	58	Retired	Married	Secondary school	MI	3
P3	Female	22	Employee	Single	Secondary school	Femur fracture	6
P4	Male	78	Farmer	Married	Primary school	Diarrhea	4
P5	Male	62	Self-employment	Married	Primary school	Cerebral hemorrhage	4
P6	Male	48	Small business	Married	Primary school	Head trauma	4
P7	Male	67	Retired	Married	Primary school	Femur fracture	4
P8	Female	35	Housewife	Married	Primary school	Chronic renal failure	10
P9	Male	52	Self-employment	Married	Illiterate	COPD	3
P10	Female	70	Retired	Single	Associate degree	COPD	7
P11	Male	57	Farmer	Married	Primary school	MI/GIS bleeding	5
P12	Male	62	Farmer	Married	Primary school	COPD	4
P13	Male	62	Chef	Married	Primary school	COPD	3

COPD: Chronic Obstructive Pulmonary Disease; MI: Myocardial Infarction; GIS: Gastrointestinal System.

**Table 2:** Categories extracted from the “intensive care experiences of the patients” theme.

Categories	P1	P2	P3	P4	P5	P6	P7	P8	P9	P10	P11	P12	P13
Thoughts about physical care	X	X	X	X	X	X	X	X	X	X	X	X	X
Thoughts about receiving psychological and social support	X	X	X	X	X	X	X	X	X	X	X	X	X
Thoughts about environmental factors	X		X	X	X	X		X		X		X	X
Thoughts about feeling safe	X	X	X		X	X	X	X	X	X	X	X	X
Thoughts about not feeling safe				X									
Thoughts about factors that increase anxiety	X	X	X	X	X	X	X	X	X	X	X	X	X
Topics of interest	X	X	X		X		X	X	X	X	X	X	X
Thinking about being out of intensive care		X	X			X	X	X	X		X	X	
Thinking about not being out of intensive care	X			X	X			X		X			X
Factors that increase pain	X	X	X	X	X	X	X	X	X	X		X	X
Communication with a healthcare professional	X	X	X	X	X	X	X	X	X	X	X	X	X
Suggestions about communication				X						X	X		
Suggestions about care		X			X			X		X		X	X
Effective methods of coping with stress	X	X	X	X	X	X	X	X	X	X		X	X
Ineffective methods of coping with stress											X		
Thoughts about spiritual care				X			X			X			X

## Data Availability

The data presented in this study were obtained through interviews with patients. Due to privacy and ethical restrictions, the data are not publicly available.
